# Management of Distal Femur Fractures: Replacement Versus Surgical Fixation Versus Conservative Management

**DOI:** 10.7759/cureus.45333

**Published:** 2023-09-16

**Authors:** Jamie C Routledge, Oladimeji Bashir, Mohamed Elbeshbeshy, Ahmed Y Saber, Adeel Aqil

**Affiliations:** 1 Orthopaedics and Trauma, Huddersfield Royal Infirmary, Huddersfield, GBR

**Keywords:** distal femur fracture management, conservative vs surgical management, surgical fixation, complex distal femur fracture, distal femur fracture

## Abstract

Introduction: Distal femur fractures are a frequently encountered injury, especially among the ageing population. Previous studies have identified that these fractures can be managed with a variety of methods and techniques which has led to an ongoing debate and investigation to decipher the optimal approach to manage these fractures.

Aim: The purpose of this study was to compare outcomes of patients managed with either distal femur replacements (DFRs), surgical fixation (SF) or conservative management. Outcomes measured included length of hospital stay, readmission rates, 30-day mortality and Oxford Knee Score.

Methods: A retrospective review was conducted, of patients admitted with distal femur fractures between June 2020 and October 2022 at Huddersfield Royal Infirmary Hospital. Patients with both native and peri-prosthetic joints were included. All patient's medical data, including imaging and operative records, were reviewed.

Results: A total of 42 patients were identified. There were six males and 36 females with a mean age of 78 years, a median age of 76 and a range of 35-102 years. Of these fractures, 15 were peri-prosthetic, and 27 were native joints. Of the patients, 30 had an SF, five had a DFR and the remaining seven were conservatively managed. Those managed with an SF had an average length of stay of 18 days, an Oxford score of 24 and two patients were readmitted within 30 days of discharge. For the DFR, the average length of stay was 16 days, an Oxford score of 22 and no patients were readmitted within 30 days. For the conservatively managed patients 21 days, an Oxford score of 25 and two patients were readmitted within 30 days of discharge. There was no 30-day mortality across all groups.

Conclusions: From our study, we can conclude that patients who managed with a DFR had the shortest length of stay in a hospital and the lowest readmission rates when compared to alternative management techniques. There was minimal difference found between the Oxford scores between all three groups. This study shows that DFR can be a safe and reliable strategy to manage distal femur fractures. Additional research is required to further compare the outcomes of these different methods of repair.

## Introduction

Distal femoral fractures are complex injuries that represent 3% to 6% of all femoral fractures and require careful consideration in terms of management strategies [1]. Current management options include distal femur replacement (DFR), surgical fixation (SF), or conservative management. However, the optimal approach for managing these fractures remains a topic of ongoing debate and investigation [2].

SF involves utilising techniques such as locking plates or retrograde intramedullary nails and is the most commonly employed surgical treatment for distal femoral fractures [3]. In studies that compared various methods of internal fixation, researchers found differing degrees of success depending on a number of criteria, including the type of implant used, the surgical procedure performed, and the rehabilitation protocols followed [4,5]. Complications such as non-union, malunion, knee stiffness, and compromised function can occur, highlighting the need for further research to optimise surgical fixation techniques [4,5].

Studies have shown that DFR has emerged as an alternative treatment option for distal femoral fractures, offering advantages such as immediate postoperative weight bearing and reduced risk of non-union, malunion, fixation failure, and post-traumatic arthritis [6]. Research studies evaluating the outcomes of DFR have demonstrated promising results, showing improved functional outcomes and patient satisfaction [6,7,8]. However, the role of DFR in the management of distal femur fractures, especially in the elderly population, requires further investigation and understanding.

Conservative management, including non-weight bearing, bracing, or casting, may be considered for select patients with distal femoral fractures. Although conservative management eliminates the risk and complications associated with surgery, it is associated with a longer period of immobilisation, an increased risk of complications such as deep vein thrombosis and pneumonia and the possibility of delayed healing or malunion [9]. It is important to have a thorough understanding of the outcomes that may be expected from conservative therapy in comparison to those that can be expected from surgical procedures when considering which treatment option would be best for a patient. The purpose of this retrospective study was to evaluate the outcomes of distal femoral fractures treated with DFR, SF, and conservative management providing valuable insights into the effectiveness of each modality.

## Materials and methods

For this study, authors identified 42 patients who had been admitted to either Huddersfield Royal Infirmary Hospital or Calderdale Royal Hospital between June 2020 and October 2022. All patients who had a distal femoral fracture were included in the study. Both native and peri-prosthetic distal femoral fractures were included. All methods of treatment for distal femoral fractures were considered in the analysis. This includes all types of surgical fixation techniques performed by our orthopaedic surgeons, as well as those managed with a conservative approach. For this study, the surgical techniques consisted of either an SF or a DFR. For the SF, this included all types of internal fixation methods used such as an open reduction internal fixation or an intramedullary nail.

In this study, the mechanism of injury was not considered during data collection. The aim of the study was not to focus on the cause of the fractures but rather on the management and outcomes depending on the treatment used for the patients with a distal femur fracture. For this data collection, the medical records and imaging for each patient were reviewed. The study considered various outcome measures, including each patient’s length of stay in the hospital, any readmission rates within 30 days of initial treatment and if there were any mortalities within 30 days of initial treatment. Comparing these variables can help to indicate complications encountered or the effectiveness of the initial treatment.

We also wanted to try and compare the level of function and pain the patients experienced following either their operation or conservative management. To do this, we used the Oxford Knee Score which is a validated questionnaire that assesses the function and discomfort associated with knee injuries or surgeries. It consists of twelve questions giving a total score out of 48, a higher number indicates a better score. To implement this, patients were contacted by telephone post-operatively to obtain their scores. Patients who were deceased or found not to have capacity were excluded from the Oxford Knee Score assessment. This means that patients who were unable to provide responses or had passed away during the follow-up period were not included in the functional outcome assessment. These measurements provide insights into the overall management and functional outcomes of patients with distal femoral fractures.

## Results

Of the 42 patients identified, our cohort consisted of six males and 36 females with a mean age of 78 years (range 35-102 years). Of these patients, 15 had a peri-prosthetic fracture, whilst the remaining 27 had fractures of native joints.

**Figure 1:**
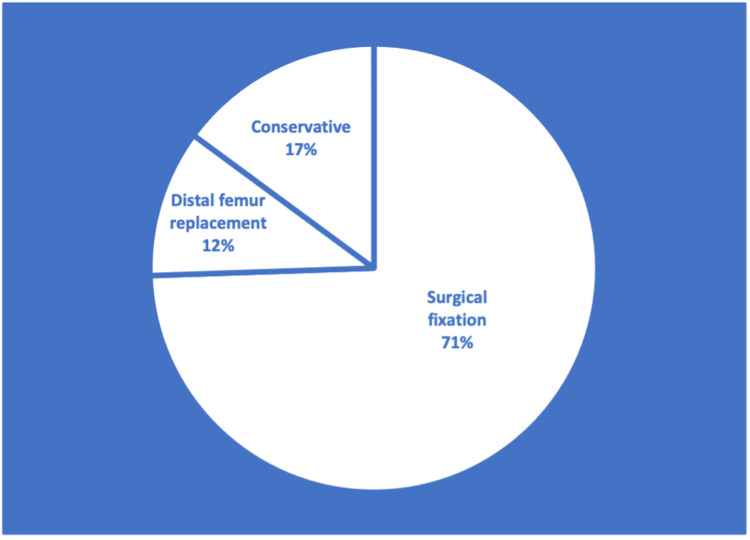
Pie chart to show the distribution of management techniques

Surgery to repair the fractured distal femur was performed on 35 patients (83%) whilst the remaining seven patients (17%) were conservatively managed. Of these 35 patients who had an operation, SF was performed on 30 patients (86%) and five patients (14%) had a DFR. Percentages are illustrated in Figure 1. The average age of those patients managed with SF was 75 years, for the DFR it was 81 years and for conservatively managed individuals it was 90 years. 

Of the 30 patients (71%) that underwent an SF, 10 of these were peri-prosthetic fractures and 20 were native joint fractures. Four of these patients were male and 26 were female. These individuals stayed in the hospital for an average of 18 days, with the length of their stays ranging anywhere from three to 45 days. The average Oxford score for these 23 patients was 24, with the score ranging anywhere from 8 to 39. We were unable to collect the scores from seven of the patients because some of the patients had either passed away or were unable to answer the questions due to diminished mental capacity. Only two patients (7%) from this group required readmission to the hospital during the first 30 days. One of these patients was readmitted 9 days following discharge due to wound dehiscence and required a wound exploration and washout. The second patient was readmitted 27 days following discharge due to a chest infection. There were no deaths within the first 30 days.

**Figure 2:**
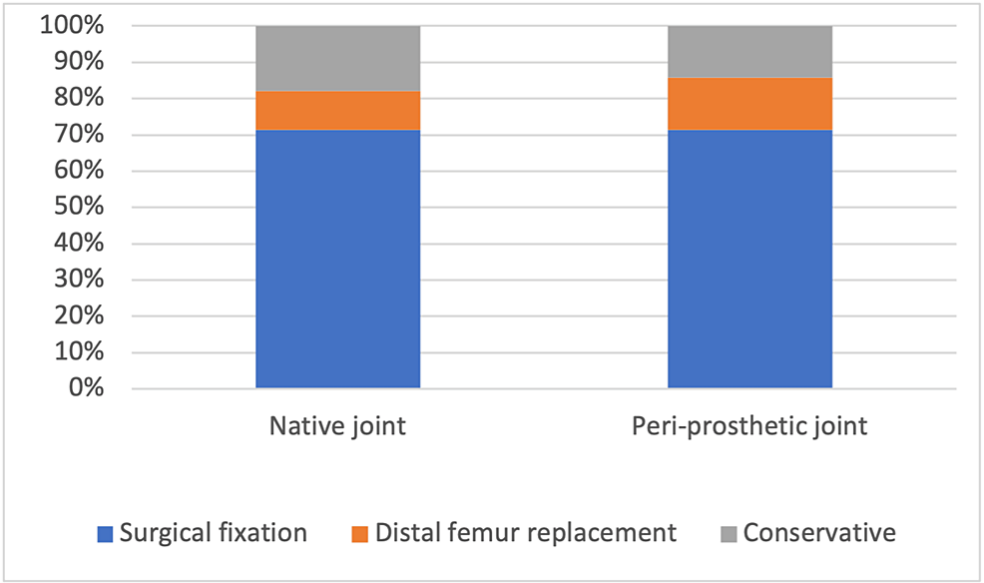
A graph to compare the management technique used depending on whether the joint was native or peri-prosthetic

Of the five patients (12%) that were managed with a distal femur replacement, two of these were peri-prosthetic fractures whilst the remaining three were from native joints. There were two male patients in this group, while the remaining three patients were female. The length of stay for these patients ranged anywhere from 5 to 24 days, with an average of 16 days. These individuals had an Oxford Knee Score that ranged anywhere from 1 to 46, with an average of 22. There was no mortality or readmission of patients within the first 30 days, and no patients died within the first 30 days. The management technique used depending on whether the joint was native or peri-prosthetic is illustrated in Figure 2.

Of the seven patients (17%) that were managed conservatively, two of these were peri-prosthetic fractures and five were native joints. All seven of these patients were female. The length of stay ranged from 3 days to 67 days, with an average of 21 days. The range of the Oxford scores for three of the patients was from 4 to 44, with the average score being 25. There was no mortality during the first 30 days for any of these patients. Two (7%) of the patients from this group were readmitted to the hospital within 30 days. One patient was readmitted 27 days following discharge due to a new onset of confusion and urinary tract infection. The second patient was readmitted one day after discharge due to ongoing mobility issues following the fracture. A comparison of the results from our study is illustrated in Table 1.

**Table 1:** Comparison of results from our study between distal femur replacement, surgical fixation or conservative management

Variable measured	Distal femur replacement	Surgical fixation	Conservative
Mean age (years)	81	75	90
Mean hospital stay (days)	16	18	21
30-day mortality	0	0	0
30-day readmission	0	2	2
Mean Oxford Knee Score	22	24	25

## Discussion

Due to an increase in the elderly population, distal femoral fractures, particularly peri-prosthetic fractures, are becoming an increasingly regular occurrence. These fractures, particularly intra-articular fractures present operative challenges for physicians and can result in significant morbidity for elderly patients [10]. The decision on which surgical technique, internal fixation, or a DFR, should be used remains controversial [11]. There have been studies that have shown that the fixation technique preference of the surgeon, as determined by which surgery they felt more experienced to perform, can have an effect on the technique that is performed [12]. 

The management of distal femoral fractures will depend on a variety of criteria, such as the age of the patient, the fracture pattern, the patient’s functional state and comorbidities [13]. There are several different approaches that can be taken to manage a distal femoral fracture. These approaches include conservative therapy, external fixation, and internal fixation including intramedullary nails and DFR [14]. DFR offers arthroplasty as a management option for patients presenting with these fractures. This technique allows for early mobilisation and can allow patients to return to their pre-operative functional status [6]. In our research, we found that patients managed with a DFR had the shortest length of stay in the hospital and the lowest readmission rates when compared to the other surgical techniques. Our results were consistent with other previous studies which have also shown DFR to have superior outcomes [15-18]. 

From our data, we found that the Oxford Knee Score was higher for the SF compared to the DFR. This indicates that these patients had a better post-operative functional status. However, we were unable to compare them to their pre-operative state. We also found that although our DFR patients had the shortest length of stay in the hospital of 16 days, the internal fixation patients were not too dissimilar with an average stay of 18 days. There have been many studies to compare the difference in outcomes between either SF or DFR for the management of distal femur fractures for both native and peri-prosthetic joints. Some of these studies have shown when comparing length of hospital stays, complication rates and functional outcomes that there is no difference between the two techniques used [2,3,15,19,20]. 

There have also been studies to show that despite initial management with fixation, due to complications these patients have had to subsequently require a DFR as a revision procedure [14]. The majority of distal femoral fractures tend to require operative management; however, they can still be managed conservatively. Indications for this include non-ambulatory patients, patients with multiple comorbidities that preclude operative fixation and nondisplaced fractures [20]. However, it has been shown in studies that those conservatively managed had a high mortality rate compared to those who underwent surgical fixation [21]. In our study, the conservatively managed patients had the highest average age and had the longest stay in the hospital. This result was expected as it is usually the more elderly patients that are more likely to have more comorbidities. Interestingly, these patients had the highest Oxford Knee Score result out of the three groups. Table 2 illustrates findings from other studies comparing the length of hospital stay following either SF or DFR.

**Table 2:** Comparison of our study with other studies

Cohort	Surgical fixation length of stay	Distal femur replacement length of stay
Our study	18	16
Hart et al. [10]	7.5	7.3
Tandon et al. [18]	32	9
Hoellwarth et al. [22]	6	5
Atrey et al. [23]	-	18.8

The Oxford Knee Score is a devised questionnaire most commonly used to assess patient outcomes following a joint replacement; however, it can also be used to evaluate other interventions like the treatment of fractures [24]. The Oxford Knee Scoring system can be used to not only assess symptoms but also the function of the knee [25]. From this study, we found that the patients managed with the SF and DFR had a similar Oxford Knee Score, with SF having a slightly higher score. Previous studies have also shown that SF and DFR have similar functional outcomes [15]. In this study, our conservatively managed patients had the best Oxford Knee Score suggesting they have the best functional outcomes and satisfaction. Previous studies have shown that those managed conservatively do not have as good a functional result when compared to those managed with surgery, so this is an area that needs further research [26].

Limitations

As we were unable to obtain Oxford Knee Scores for some of the patients due to either cognitive impairment or death, this can be considered as one of our limitations, as it reduced our comparative data. Due to the time period data was collected the Oxford Knee Scores were obtained at different stages post-operatively, which could have an impact on the patient’s score given. However, we allowed for a minimum of 9 months between obtaining the score and when the patient was initially admitted with the fracture. 

## Conclusions

According to the findings of our study, a DFR appears to be a successful procedure that can provide favourable outcomes. Patients who had a DFR spent the least amount of time in the hospital overall and had the lowest rate of readmission when compared to alternative management options. However, patients managed with DFR had the lowest Oxford Knee Score when compared to the other two groups. This study demonstrates that DFR can be an approach that is both safe and reliable. However, additional research is required to further understand the optimum management for distal femoral fractures.
